# Diagnostic Challenges and Treatment of Concurrent Toxoplasmosis and Disseminated Cryptococcus in an Immunocompromised Patient

**DOI:** 10.1155/crdi/9917703

**Published:** 2025-06-21

**Authors:** Jeffrey Valencia Uribe, Ann-Katrin Valencia, Brian Nudelman, Nicole Nudelman, Dante P. Melendez Lecca

**Affiliations:** ^1^Internal Medicine, Memorial Healthcare System, Pembroke Pines, Florida, USA; ^2^Division of Infectious Disease, Memorial Healthcare System, Pembroke Pines, Florida, USA

## Abstract

**Background: **Co-infection with disseminated cryptococcosis and toxoplasma encephalitis is rare but presents significant diagnostic and therapeutic challenges, particularly in severely immunocompromised patients. This case study highlights the complexities involved in managing such dual infections.

**Case Presentation:** We describe a 43-year-old Hispanic male with Stage IV EBV-positive diffuse large B-cell lymphoma and hemophagocytic lymphohistiocytosis who presented with progressive weakness and altered mental status. Initial brain MRI revealed multiple enhancing lesions. Diagnostic tests for cryptococcosis and toxoplasma were inconclusive; however, a positive cryptococcal antigen test, new lung nodules, and potential central nervous system involvement suggested possible disseminated cryptococcosis. Diagnosis of cryptococcal meningoencephalitis could not be confirmed due to negative CSF cultures.

**Management and Outcome:** Despite initiating treatment with amphotericin B and flucytosine for suspected cryptococcosis, the patient's condition did not improve. Initial Karius and CSF PCR tests for Toxoplasma were negative. A subsequent brain biopsy, however, confirmed toxoplasmic encephalitis. Treatment was adjusted to intravenous Trimethoprim/Sulfamethoxazole for toxoplasmosis, with continued fluconazole for cryptococcosis. The patient exhibited significant clinical improvement with this revised therapy.

**Conclusion:** Diagnosing concurrent cryptococcal and toxoplasma infections is challenging due to overlapping clinical symptoms and variability in test sensitivities. This case underscores the need for a comprehensive diagnostic approach and the critical role of brain biopsy when other diagnostic methods, such as Karius testing and CSF PCR, are inconclusive. Prompt empirical treatment based on clinical suspicion, with subsequent treatment adjustments guided by clinical response and follow-up assessments, is essential for effective management.

## 1. Introduction

Co-infection with disseminated cryptococcosis and toxoplasma encephalitis is extremely rare, even among immunocompromised patients. Those who are affected by both conditions typically have very low CD4 counts, often below 100 cells/Μl [1]. Patients with this dual infection generally face more prolonged hospitalizations, frequent admissions to intensive care units, and increased in-hospital mortality rates. Managing these individuals presents significant challenges due to the complexities involved in diagnosing both infections and the need for meticulous coordination of antifungal and antiparasitic therapies [1].

Cryptococcus is transmitted through inhalation of fungal spores from the environment. The primary pathogens are *Cryptococcus neoformans* and *Cryptococcus gattii*, both of which possess a polysaccharide capsule that aids in evading the host immune response [2]. While Cryptococcus primarily targets the lungs and central nervous system, it can less commonly infect the skin, bone, eyes, and prostate. In severely immunocompromised individuals, such as those with AIDS, diabetes, transplant recipients, and patients on prolonged steroid therapy, the infection can affect virtually any organ. These individuals are particularly vulnerable due to their weakened immune systems, which allow the fungus to proliferate and spread beyond the initial site of infection. In contrast, healthy individuals often experience asymptomatic initial infections.

The most common clinical manifestation of disseminated cryptococcosis is meningoencephalitis. Symptoms typically include headache, altered mental status, confusion, stiff neck, nausea, vomiting, fever, and malaise [3]. Ocular involvement, such as papilledema and uveitis, occurs in about 40% of patients [2]. Though less common, some patients may also report symptoms like cough, dyspnea, and skin rashes [3].

Toxoplasmosis is commonly acquired through the consumption of raw or undercooked meat, unwashed raw vegetables and fruits, or exposure to cat feces. *Toxoplasma gondii* can form cysts in the brain, which may reactivate in immunocompromised individuals, leading to active infection and subsequent brain damage [4]. Clinical manifestations of toxoplasmosis encephalitis include headache, confusion, fever, and lethargy [4]. Additional possible symptoms can encompass cranial nerve disturbances, sensory abnormalities, cerebellar signs, movement disorders, and neuropsychiatric symptoms. The most frequent focal neurological signs are hemiparesis and speech abnormalities. Seizures are observed in up to 30% of patients, while focal neurological deficits are present in up to 70% [4].

A challenging case is presented involving a diagnosis of toxoplasmosis encephalitis with concomitant disseminated cryptococcus in an immunocompromised patient with numerous brain-enhancing lesions.

## 2. Case Presentation

A 43-year-old Hispanic male with a history of autism spectrum disorder, supraventricular tachycardia, Stage IV Epstein–Barr virus (EBV)-positive diffuse large B-cell lymphoma, not otherwise specified (DLBCL-NOS), with bone marrow involvement and hemophagocytic lymphohistiocytosis (HLH), on viral prophylaxis presented to the emergency department with progressive weakness and recurrent falls secondary to altered mental status.

Four months prior to the presentation, he was diagnosed with DLBCL-NOS and secondary HLH. The diagnosis of HLH was established using an H score of 259, indicating a greater than 99% probability of HLH. The patient was found to be interleukin-2 receptor positive, meeting criteria for treatment with etoposide. He was subsequently initiated on DA-EPOCH (etoposide 200 mg IV, prednisone 140 mg PO, vincristine 0.96 mg IV, cyclophosphamide 3 g IV, doxorubicin 40 mg IV). Following five cycles of DA-EPOCH, he achieved complete remission.

**Figure 1:**
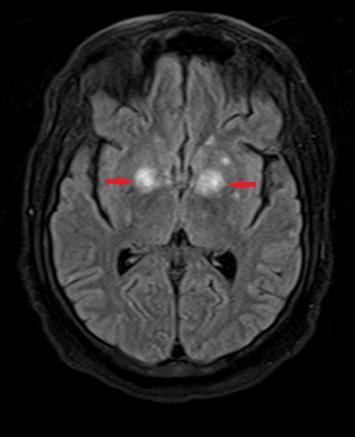
MRI of the brain without contrast obtained 3 months prior to admission revealed numerous subcentimeter-enhancing intra-axial lesions scattered throughout the cerebral and cerebellar hemispheres. The most prominent lesions were located in the thalami and bilateral basal ganglia (red arrows).

One month after initiating chemotherapy, the patient developed new-onset seizures. At that time, an MRI of the brain without contrast (Figure 1) revealed multiple enhancing lesions, with the largest located in the thalami and bilateral basal ganglia. Lumbar puncture results were negative for cytology, cancer, and infection, including a negative CSF toxoplasma PCR, which suggested that the seizures were likely due to intrathecal methotrexate or chemotherapy-induced effects.

**Figure 2:**
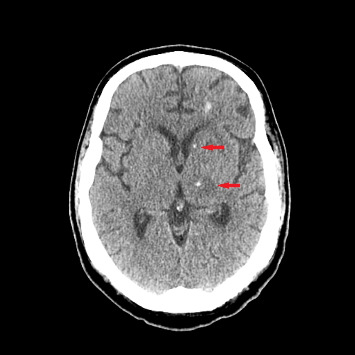
CT of the brain showed scattered hyperdense lesions with surrounding edema (red arrows). These findings are similar to prior MRI brain.

Upon the current admission to the emergency department, the patient's vital signs were as follows: blood pressure, 116/61 mmHg; temperature, 36.3°C; heart rate, 129 bpm; and respiratory rate, 99 breaths per minute. The physical examination revealed that the patient was alert, oriented only to self, and able to follow commands but exhibited limited language skills, dysarthria, and hypophonia. Neurologically, the patient demonstrated significantly decreased strength: 1/5 in right hip flexion, knee flexion, knee extension, dorsiflexion, and plantar flexion; 2/5 in left hip flexion, knee flexion, and knee extension; and 0/5 in left dorsiflexion and plantar flexion. Reflexes were 1+ in both upper and lower extremities. Laboratory results showed anemia with a hemoglobin level of 9.7 g/dL and hyponatremia with a sodium level of 132 mEq/L. Blood and urine cultures were negative. Plasma EBV PCR was undetectable. An initial CT scan of the brain without contrast (Figure 2) showed edema in the thalami and bilateral basal ganglia, with no significant change compared to the previous MRI.

**Figure 3:**
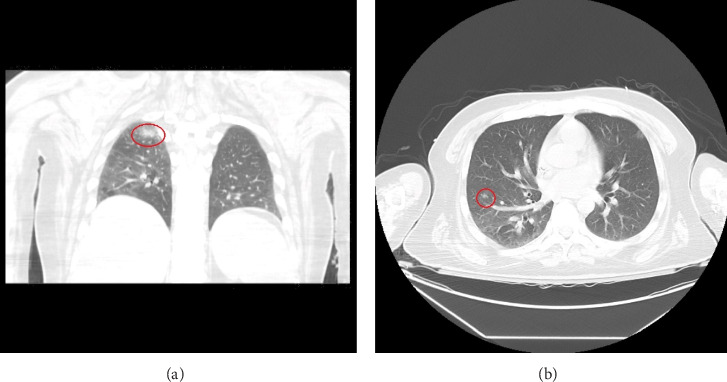
CT of the chest without IV contrast revealed a small area of subsegmental pneumonia in the superior segment of the right lower lobe ((a) encircle in red), along with multiple subcentimeter lung nodules ((b) the largest 6.1 mm nodule is encircled in red), accompanied by a small patch of ground-glass opacities in the right posterior lung base.

An infectious work-up for opportunistic infections was conducted. Relevant findings included elevated serum Toxoplasma IgG at 46 IU/mL, a positive cryptococcal antigen test with a titer of 1:2560, and a negative Karius test. To investigate potential other sites of infection, a CT scan of the chest without IV contrast (Figure 3) was performed, which revealed possible small pneumonia in the right lower lobe and multiple subcentimeter nodules bilaterally, raising concerns for opportunistic infections. The patient was initiated on treatment with flucytosine and amphotericin B due to suspected disseminated cryptococcosis. Follow-up cryptococcal antigen tests on days 2 and 3 after the initial positive result showed decreased titers of 1:80 and 1:40, respectively.

**Figure 4:**
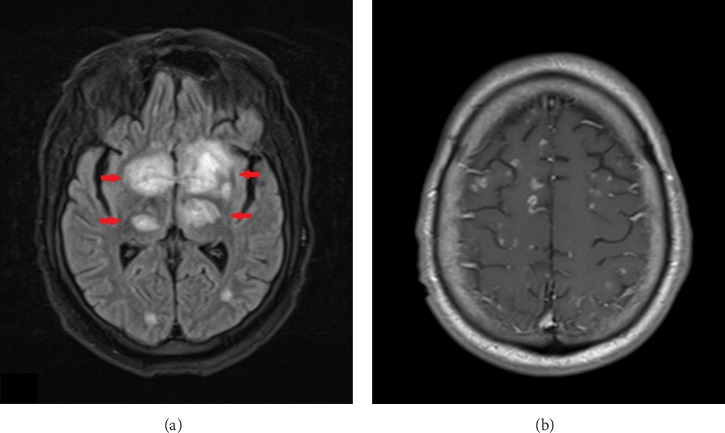
MRI of the brain without contrast shows an increase in both the number and size of the previously noted enhancing lesions throughout the brain parenchyma ((a) and (b)), affecting both the cerebral and cerebellar hemispheres as well as the brainstem. The largest lesions are located in the basal ganglia and thalami ((a) red arrows).

MRI of the brain (Figure 4) revealed enlarging enhancing lesions with increased edema. An MRI of the spine without contrast showed no acute abnormalities. Initial evaluation by neurosurgery concluded that the patient was not a candidate for a brain biopsy due to the lesion's location. Cerebral edema was managed with IV steroids, and the patient was started on meropenem and vancomycin to cover potential *Nocardia* and bacterial pyogenic abscesses. A subsequent lumbar puncture revealed negative results for the meningitis/encephalitis panel, CSF flow cytometry, and cytology, which only showed reactive lymphocytes without evidence of malignancy. The CSF was also negative for cryptococcal antigen and Toxoplasma PCR, and showed no growth on gram stain or fungal culture.

**Figure 5:**
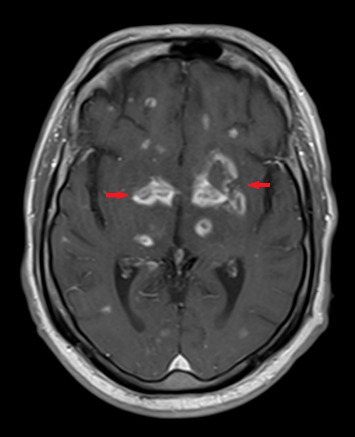
MRI of the brain with and without contrast reveals a worsening condition with an increase in both the number and size of lesions. There are numerous peripheral enhancing and nodular lesions throughout the cerebral and cerebellar hemispheres as well as the brainstem. The largest lesions (red arrows) are located in the deep gray nuclei, accompanied by increased edema.

Despite 2 weeks of treatment, the patient's mental status remains poor, and there has been no clinical improvement. A repeat CT scan of the chest revealed resolution of the lung nodules. However, a follow-up MRI of the brain with and without contrast (Figure 5) demonstrated the emergence of numerous new enhancing lesions and increased edema.

Since the diagnosis of disseminated cryptococcal infection was not definitively established—due to negative cryptococcal PCR in the CSF, a negative Karius test, and lack of response to amphotericin B and flucytosine—the suspicion that the brain lesions were caused by cryptococcus decreased. Consequently, amphotericin B and flucytosine were discontinued, and treatment was transitioned to fluconazole 800 mg orally daily for 8 weeks of consolidation therapy, followed by 200 mg daily for maintenance therapy for probable disseminated cryptococcosis. Antibiotics were added to address a possible pyogenic abscess, though this was considered less likely due to the poor response. CNS lymphoma was considered a differential diagnosis; however, a recent PET scan indicated improvement in disease, and a recent CSF analysis revealed no lymphocytes compatible with lymphoma. Toxoplasmosis remained a consideration due to positive serum Toxoplasma IgG, but a definitive diagnosis was elusive as both the Toxoplasma PCR in the CSF and the initial Karius test was negative.

Two to 3 weeks after admission, the patient's altered mental status worsened, and he became unable to swallow. Neurosurgery was reconsulted for a brain biopsy. A right frontal craniotomy with stealth navigation for lesion resection and biopsy was performed. While awaiting biopsy results, the second Karius test was positive for Toxoplasma gondii. Brain tissue culture revealed one colony of *Staphylococcus aureus*, prompting a switch to Nafcillin for 3 weeks to address a possible abscess. Immunostaining of the brain biopsy was positive for Toxoplasma, confirming the diagnosis of Toxoplasma encephalitis. Treatment with intravenous Trimethoprim/Sulfamethoxazole was initiated, as the patient was NPO due to his altered mental status.

**Figure 6:**
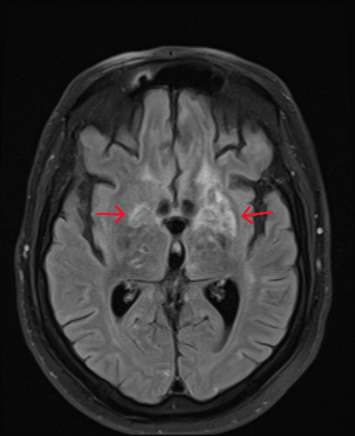
MRI of the brain with and without contrast demonstrates that many of the previously enhancing lesions now show no enhancement. The persisting lesions (red arrows) exhibit reduced enhancement and edema compared to earlier imaging, indicating a partial response to therapy.

One month after starting treatment, the patient demonstrated clinical improvement, resolving altered mental status and increased wakefulness and interaction, suggesting a positive response to therapy. A follow-up MRI of the brain with and without contrast (Figure 6) showed a reduction in lesions and edema. The patient underwent fiberoptic endoscopic evaluation of swallowing, in which he was determined to have moderate oral motor and pharyngeal dysphagia with recommendations to trial oral intake. Trimethoprim/Sulfamethoxazole was transitioned to oral administration with an induction dose of 400 mg/2000 mg BID for an additional 4 weeks.

The patient experienced a complex hospital course, including a multidrug-resistant Proteus UTI, which was treated with ertapenem, and Strongyloides infection, managed with ivermectin. During hospitalization, oncology evaluations confirmed remission, as CT scans of the neck, chest, abdomen, and pelvis showed no active lymphoma. The patient's condition progressively improved; however, he required substantial assistance during physical therapy sessions. It was determined that he had experienced a significant functional decline and impaired mobility. Consequently, he was discharged to a skilled nursing facility to receive specialized care and support.

Two weeks postdischarge, the patient was assessed in the outpatient infectious disease clinic. He has returned to his baseline cognitive function but continues to have some motor deficits. The plan is to maintain treatment with Bactrim double strength daily for toxoplasmosis and fluconazole 200 mg daily for cryptococcal prophylaxis.

## 3. Discussion

The differential diagnosis for brain-enhancing lesions in immunocompromised patients includes progressive multifocal leukoencephalopathy, toxoplasmosis encephalitis, cryptococcal meningoencephalitis, cytomegalovirus encephalitis, fungal infections (e.g., aspergillosis and mucormycosis), primary central nervous system lymphoma, metastatic disease, neurosarcoidosis, and autoimmune encephalitis [5]. Diagnosing concurrent infections of disseminated cryptococcus and toxoplasmosis encephalitis is challenging due to their overlapping clinical presentations, which include headaches, fever, seizures, and neurological deficits [1]. In this immunocompromised patient, the initial symptom was a seizure that occurred 3 months prior to his admission. The initial MRI of the brain (Figure 1) revealed enhancing lesions, but other infectious workups, including serum cryptococcal antigen, CSF analysis, and cultures, were negative. This suggested that the enhancing lesions might be attributable to intrathecal methotrexate or chemotherapy rather than an infectious etiology.

Seizures induced by intrathecal methotrexate are documented in the literature, particularly in patients receiving high doses or those with pre-existing CNS pathology. Such seizures can present with leukoencephalopathy, focal enhancing lesions, or cortical abnormalities [5]. Despite discontinuing intrathecal methotrexate, the patient's deteriorating mental status and follow-up MRI of the brain (Figure 4) showing an increase in the size and number of enhancing lesions with worsening edema suggested the need to consider alternative diagnoses.

Neuroimaging of cryptococcal encephalitis via MRI can reveal a range of findings, including single or multiple lesions with or without enhancement or edema, hydrocephalus, dilated perivascular spaces, pseudocysts, and basilar meningeal enhancement [6–8]. Commonly affected areas include the basal ganglia, thalamus, and cerebellum. In contrast, toxoplasmosis typically presents on MRI or CT of the brain as multiple ring-enhancing lesions, often located in the basal ganglia, thalami, or corticomedullary junction [9]. While these imaging features suggest toxoplasmosis encephalitis, they are not definitive and must be corroborated with additional diagnostic tests. In this patient, the MRI revealed the most prominent enhanced lesions in the thalami and basal ganglia, which are characteristic of both toxoplasmosis and cryptococcal infections.

Further evaluation showed cryptococcal antigen titers greater than 1:512 and the presence of lung nodules, raising suspicion for disseminated cryptococcosis. However, a definitive diagnosis of cryptococcal meningoencephalitis requires a positive culture for the organism in the CSF, and diagnosis of disseminated cryptococcosis is typically confirmed by positive blood culture or culture from at least two different sites [3]. The repeat CT scan of the chest demonstrated the resolution of the lung nodules, suggesting an adequate response to treatment for pulmonary cryptococcosis. Despite this, the patient's clinical condition did not improve after 2 weeks of treatment, prompting further investigation.

A brain tissue biopsy ultimately confirmed toxoplasmosis encephalitis. Diagnosis of Toxoplasma infection can be achieved through indirect serological methods and direct detection techniques, depending on the patient's immune status. Indirect detection is done via Toxoplasma IgG or IgM antibodies. Elevated IgG levels indicate past exposure and a potential risk of reactivation. However, IgM may be absent even during active disease in immunocompromised patients, making it a less reliable marker. A negative IgM result may help rule out a recent infection.

Toxoplasmosis was initially considered less likely in our differential diagnosis, given the negative results from the initial Karius and CSF tests. Although the Karius test can aid in the noninvasive diagnosis of opportunistic infections, it cannot serve as a definitive rule-out test. A study involving 106 patients with hematological malignancies suspected of pulmonary invasive fungal disease compared plasma samples from bronchoalveolar lavage using the Karius test to standard diagnostic methods. The study found that the Karius test had a sensitivity of 44% and a specificity of 97% compared to conventional diagnostic methods [10].

The initial CSF PCR for *Toxoplasma gondii* was negative. Direct detection of *T. gondii* is performed via PCR for its DNA in various body fluids, including blood, urine, and CSF. For diagnosing toxoplasmosis encephalitis in immunocompromised patients, PCR of the CSF is crucial. When contamination is ruled out, PCR results typically demonstrate near-perfect specificity, ranging from 94.4% to 100%, and a positive predictive value approaching 100%. However, multiple studies have reported a broad sensitivity ranging from 64% to 100% [11–14]. Thus, a negative PCR result does not entirely exclude toxoplasmosis encephalitis. Sensitivity may be affected by factors such as pathogen load and prior treatment, with low pathogen load potentially leading to false-negative results. False negatives can also occasionally occur due to the prozone phenomenon, where high antibody titers interfere with the visualization of antigen-antibody complex agglutination; however, this is unlikely in this case [15]. A definitive diagnosis can be made through a biopsy of brain tissue to identify *T. gondii* tachyzoites or cysts, which can be particularly useful when PCR and clinical findings are inconclusive.

Another potential consideration was the possibility of a *Staphylococcus aureus* pyogenic brain abscess. *Staphylococcus aureus* accounts for 10%–20% of brain abscess cases in the general population and is commonly associated with cranial trauma or endocarditis [16]. Despite this, our patient did not exhibit these risk factors. A single colony of *Staphylococcus aureus* in the brain tissue could be considered a contaminant; however, treatment was warranted due to the patient's poor overall clinical status.

The treatment for disseminated cryptococcosis, cryptococcal meningitis, and severe isolated pulmonary cryptococcosis typically involves a multi-phase approach. The initial induction therapy consists of liposomal amphotericin B at 3–4 mg/kg daily, combined with flucytosine at 25 mg/kg four times a day for 2 weeks. This is followed by consolidation therapy with fluconazole, dosed at 400–800 mg daily for 8 weeks. After consolidation, maintenance therapy with fluconazole at 200 mg daily is recommended for at least 12 months [5].

First-line treatment for toxoplasmosis encephalitis in immunocompromised patients typically involves a combination of pyrimethamine, sulfadiazine, and leucovorin [17]. The initial oral dose of pyrimethamine is 200 mg once, followed by a weight-based regimen. For patients weighing 60 kg or less, the treatment consists of oral pyrimethamine 50 mg daily, sulfadiazine 1000 mg every 6 hours, and leucovorin 10–25 mg daily. For patients over 60 kg, the oral dosage is pyrimethamine 75 mg daily, sulfadiazine 1500 mg every 6 hours and leucovorin 10–25 mg daily. The initial treatment duration is typically 6 weeks. Prolonged induction therapy may be indicated in cases of delayed clinical response, severe disease, profound immunosuppression, or persistent or recurrent symptoms.

If patients cannot tolerate sulfonamides or do not respond to the first-line regimen, clindamycin 600 mg administered intravenously or orally every 6 hours can be used as an alternative to sulfadiazine [17]. There is no parenteral form of pyrimethamine for patients who cannot take oral medications. An alternative is TMP-SMX, with TMP dosed at 5 mg/kg and SMX at 25 mg/kg twice daily, which provides the sulfonamide component.

For maintenance therapy, the preferred regimen is oral pyrimethamine 25–50 mg daily, sulfadiazine 2000–4000 mg daily, and leucovorin 10–25 mg daily. However, a meta-analysis has demonstrated that TMP-SMX is a viable alternative for maintenance therapy, offering comparable efficacy with reduced toxicity and lower cost compared to pyrimethamine-based regimens [18]. Therefore, due to considerations of cost and availability, TMP-SMX DS may be used twice or once daily as an alternative. Other oral alternatives include clindamycin 600 mg every 8 hours, pyrimethamine 25–50 mg daily, and leucovorin 10–25 mg daily. Atovaquone, 750–1500 mg, administered orally twice daily, can also be used as a single agent or in combination; however, its efficacy compared to other regimens is less well-documented.

## 4. Conclusion

This case highlights the complexities in distinguishing between cryptococcal and toxoplasmosis encephalitis in immunocompromised patients, especially when initial diagnostic tests yield inconclusive results. The varying sensitivity of PCR fluid analysis, Karius testing, and serum antigen tests underscores the need for a comprehensive diagnostic approach rather than relying on a single test. Brain biopsy remains a crucial tool for diagnosis when other methods are insufficient.

Prompt empirical treatment is essential when an opportunistic infection is suspected, even before a definitive diagnosis is established. This approach should be accompanied by obtaining appropriate cultures and initiating antifungal, antiviral, or antiparasitic therapies as needed. Laboratory findings and clinical response to treatment are pivotal in guiding diagnosis and management. Ultimately, clinical improvement serves as a crucial indicator of treatment efficacy, helping to refine the diagnostic process. In this case, the patient showed significant improvement with appropriate treatment for Toxoplasma encephalitis and is being followed up as an outpatient for prophylaxis treatment and management of residual motor deficits.

## Data Availability

No datasets were generated or analyzed during the current study. Patient data was gathered from electronic health records.
